# Acknowledgement to Reviewers of *Microorganisms* in 2019

**DOI:** 10.3390/microorganisms8020155

**Published:** 2020-01-22

**Authors:** Microorganisms Editorial Office

**Affiliations:** MDPI, St. Alban-Anlage 66, 4052 Basel, Switzerland

The editorial team greatly appreciates the reviewers who have dedicated their considerable time and expertise to the journal’s rigorous editorial process over the past 12 months, regardless of whether the papers are finally published or not. In 2019, a total of 756 papers were published in the journal, with a median time to first decision of 17 days and a median time from submission to publication of 38 days. The editors would like to express their sincere gratitude to the following reviewers for their generous contribution in 2019:
Abd El Wahed, AhmedLin, Yu-HungAbdallah Abdelmohsen, MohamedLindner, MartinaAbdul-Aziz, HafizLing, MauriceAbia, Akebe Luther KingLinke, DirkAbrouk, DanisLiu, WeiAbrunhosa, LuísLiu, YangAbu-Niaaj, LubnaLo Giudice, AngelinaAccardi, LuisaLøbner-Olesen, AndersAcedo, Jeella Z.Łobocka, MalgorzataAchal, VarenyamLock, AntoniaAdam, GerhardLõhmus, AskoAdamczyk-Popławska, MonikaLomeli_Ortega, CarlosAdams, Leslie GarryŁoniewski, IgorAdhikari, Bal RamLopes, GracilianaAdhikari, RajanLoppi, StefanoAdiletta, GiuseppinaLorenz, AlexanderAgak, George WLosio, Marina NadiaAgaphonov, MichaelLosurdo, GiuseppeAgarwal, VinayakLu, XindaAggarwal, Bharat B.Lucas, RudolfAhern, PhilipLu-Chau, ThelmoAimanianda, VishukumarLuis, AnaAira Vieira, ManuelLuksiene, ZivileAkter, Yeasmin AraLuptakova, LenkaAlduina, RosaLyons, TamsinAlex, AnoopLysak, Lyudmila LysakAlias-Villegas, CynthiaMa, KesenAlifano, PietroMa, ZhengxinAlmeida, AdelaideMaccallini, CristinaAlster, Charlotte J.Maccario, LorrieAlves, VivianeMacGowan, Alasdair P.Amaretti, AlbertoMaciá-Vicente, Jose GasparAmbrosini, RobertoMackenstedt, UteAmbrosino, ElenaMacori, GuerrinoAmenabar, MaximilianoMadrid, AlejandroAmils, RicardoMaeda, AkihikoAmund, DanielMaehara, ShojiAncuceanu, RobertMaffei, GianlucaAnderson, O. R.Magiorkinis, EmmanouilAnderson, Robin CMagurno, FrancoAngelici, Maria CristinaMagyar, DonatAngione, ClaudioMahapatra, Ajit K.Anić, IvicaMaher, L. JamesAnne, JozefMaicas, SergiAnnesley, SarahMakris, AntoniosAnniballi, FabrizioMakwana, NandiniAntal, DianaMalacrinò, AntoninoAntolak, HubertMalagon, FranciscoAppelberg, RuiMalgieri, GaetanoArana, InesMan, AdrianAranda, AgustínManak, Mark M.Araniciu, CătălinMancabelli, LeonardoArchacka, MartaMancini, InesArchibald, Lennox KennethManfrin, AmedeoArciello, AngelaManjunath, ManuboluArdanuy, CarmenMankowski, MarkArdley, JulieMannazzu, IlariaArmalyte, JulijaMarc, DanielArmon, RobertMarchetti, MartaArnold, KellyMaresca, MarcArqués, Juan L.Marino, AndreanaArruda, Bailey L.Markou, NikolaosArsenault, Ryan J.Markovska, RumyanaArslan, MuhammadMárová, IvanaArthur, Terrance M.Marquart, MaryArvaniti, KostoulaMarshall, Ian P.G.Ascenzioni, FiorentinaMartienssen, MarionAspatwar, AshokMartín, Juan FranciscoAthrey, GiridharMartínez-Avilés, MartaAtienzar, PedroMartinez-Gil, LuisAtwood, DavidMartins, CarlaAubert, RachaelMartins, Gislâine A.Ausra, SipailieneMartins, IsabelAwasthi, MayankaMartinson, Guntars O.Ayad, AmiraMaruyama, ShinichiroAylward, Frank OMarzec-Grządziel, AnnaAzab, WalidMaslow, Joel N.Azam, Aa HaerumanMassari, PaolaAzman, SametMast, YvonneAzzimonti, BarbaraMasuda, SachikoBachmann, NathanMathai, PrinceBachvaroff, Tsvetan RMathew, AnujaBacosa, Hernando P.Matilla, MiguelBailey, ConstanceMatlakowska, RenataBaker, Jonathon L.Matos, PaulaBaldassarre, Maria ElisabettaMatsuki, TakahiroBaliraine, FrederickMatsuo, TakashiBallou, ElizabethMatsuoka, KazumiBalogh-Brunstad, ZsuzsannaMatsuzaki, RyoBambling, MatthewMatteucci, EnricaBanach, ArturMatthews, Jennifer L.Banerjee, SantanuMaurer-Stroh, SebastianBanerjee, SudipMauriello, FrancescoBani, AlessiaMaurya, ShailendraBaragetti, AndreaMayengbam, ShyamchandBaraldi, ElenaMayo-Prieto, SaraBarbosa, CatarinaMayumi, DaisukeBarlow, RobertMcCarthy, AlexBarnett, Stephen J.McClelland, ErinBaron, SandrineMcDermott, Suzanne M.Bartocci, PietroMcEwan, NeilBartoli, FlaviaMcFeeters, RobertBartoloni, AlessandroMcIntyre, Thomas M.Barton, Larry LMcWhorter, AndreaBartosz, GrzegorzMechaly, ArielBarzon, LuisaMegaw, JulianneBasmaciyan, LouiseMehaffy, CarolinaBastidas, Robert J.Mehariya, SanjeetBastidas-Oyanedel, Juan-RodrigoMeinersmann, Richard J.Battistoni, AndreaMelcher, UlrichBaz, MarianaMelini, FrancescaBeccari, GiovanniMelter, OtoBeck, ReljaMelton-Celsa, AngelaBecker, YvonneMenanteau-Ledouble, SimonBehzadi, PayamMenendez, EstherBej, AsimMengoni, AlessioBekhit, Alaa El-Din AMenocal, OctavioBelbahri, LassaadMenon, VipinBeld, JorisMensah-Attipoe, JacobBelenguer, AlvaroMenzel, LorenzoBell, RebeccaMesalam, AhmedBeloukas, ApostolosMetras, RaphaelleBel’skaya, LyudmilaMézes, MiklósBerckmans, BarbaraMezule, LindaBerger, BeatriceMiceli, CristinaBergholz, TeresaMickol, Rebecca L.Bermudez, Luiz E.Midelet-Bourdin, GraziellaBernstein, AnatMiele, Adriana E.Berta, GraziellaMierzejewska, JolantaBertaccini, AssuntaMihai, MaraBertelloni, FabrizioMihalca, Andrei DanielBerthold-Pluta, AnnaMilhano Santos, FátimaBertoglio, FedericoMILLEMANN, YvesBertram, RalphMiller, DarleneBesse-Hoggan, PascaleMiller, Joanna L.Besteiro, SébastienMills, Aaron L.Betekhtin, AlexanderMinami, MasaakiBeule, LukasMinamisawa, KiwamuBeumer, RijkeltMir Sanchis, IgnacioBevilacqua, AntonioMIRONESCU, MonicaBezirtzoglou, EugeniaMisguich, FabienneBezza, Fisseha AndualemMiura, NatsukoBhak, JongMiyoshi, Shin-ichiBhavsar, Amit P.Moar, WilliamBiasato, IlariaMoens, Ugo LionelBielefeldt-Ohmann, HelleMoestrup, ØjvindBiernasiuk, AnnaMohan, Ganesh Babu MalliBilin´ska, LucynaMohd Yusof, Barakatun NisakBillard, ElisabethMołoń, MateuszBisanz, JordanMonk, JonathanBitar, IbrahimMontag, DirkBjelic, DraganaMontaño, AlfredoBlaise, MickaelMonteiro, MarioBlanco-Lobo, PilarMoolhuijzen, Paula M.Blanton, CynthiaMoore, ChesterBloch, SylwiaMora-Montes, HectorBlock, Jean-ClaudeMorgan, SydneyBoaz, MonaMorgunov, IgorBocianowski, JanMori, KazuhiroBock, C. ThomasMori, MatteoBoldura, Oana-MariaMoriyama, HiromitsuBonamore, AlessandraMormann, MichaelBonnet, SylvestreMorris, MhairiBonos, EleftheriosMorrison, DouglasBopegamage, ShubhadaMorsy, MustafaBorghese, RobertoMota, L. JaimeBorghi, ElisaMottawea, WalidBortoluzzi, CristianoMoxley, Rodney A.Börve, JorunnMozejko-Ciesielska, JustynaBorza, TudorMucciarelli, MarcoBosilevac, JosephMudalige, Thilak K.Bosnea, LouloudaMuggia, LuciaBosso, LucianoMukhopadhyay, SomshuvraBouaïcha, NoureddineMulas, MaurizioBourne, Christina R.Muller, EmilieBraham Deep Singh, DhillonMüller, JoachimBrandenburg, Karen M.Müller, VolkerBratkic, ArneMunekata, PauloBrenišin, MarekMurakami, TakumiBreton, LionelMurata, TakayukiBrigido, ClarisseMurphy, BrianBrites, DanielaMurray, ShaunaBritz, Margaret L.Mustapha, AzlinBroecker, FelixMuth, TheodoreBroom, Leon J.Muth, ThiloBrosnahan, C. L.Muthu Karuppan, Mohan KumarBrown, CelesteMysliwiec, TamiBruchhaus, IrisNaganuma, TakeshiBrumm, Phillip J.Nagel, RaimundBrycki, BogumilNagpal, RavinderBuchanan, RobertNakai, RyosukeBuckel, WolfgangNakajima, MasamiBuján, NoemíNakamoto, ShingoBulatov, EmilNakatsuji, TeruakiBulmer, Mark S.Nanbo, AsukaBurkowska-But, AleksandraNanduri, RavikanthBurrascano, JosephNanjundaswamy, AnandaBurt, Sara A.Nardi, TizianaButi, MatteoNardini, ChristineButnariu, MonicaNasir, ArshanButtiglieri, GianluigiNäslund, JohanButu, AlinaNastro, Rosa AnnaCaballero, ArmandoNath, ArijitCabezas-Cruz, AlejandroNebeská, DianaCaceres, IsauraNelsen, Matthew P.Caeiro, MariaNemec, AlexandrCalcutt, MichaelNero, LuisCamacho Chab, Juan CarlosNerva, LucaCamacho, MaríaNeu, ThomasCamacho-Ruiz, Rosa MaríaNeubauer, KatarzynaCampbell, Lee AnnNg, Jason Liang PinCampbell, SaraNguewa, PaulCampos, Maria DoroteiaNguyen, Quang D.Campos, Maria Jorge GeraldesNguyen, Van KhanhCanales, RobertNiculita-Hirzel, HélèneCaneiras, CátiaNiczyporyk, Jowita SamantaCann, IsaacNiedźwiedzka-Rystwej, PaulinaCanonico, LauraNigris, SebastianoCantoral, Jesús ManuelNikoh, NaruoCao, JialanNikolich, MikeljonCapasso, RaffaeleNilsson, R. HenrikCardoso, PauloNimmagadda, AlekhyaCaro, YanisNishide, YudaiCarotti, SimoneNishiyama, MakotoCarretto, EdoardoNissinen, RiittaCarrillo, Catherine D.Niu, DongyanCarro, LorenaNoel, Samantha J.Carrouel, FlorenceNorén, TorbjörnCartmill, Donita L.Notas, GeorgeCaseys, CélineNtaikou, IoannaCass, Bodil N.Ntougias, SpyridonCastellani, GastoneNúñez-Pons, LauraCastiglione, FrancaNwaiwu, OgueriCatry, BoudewijnNychas, George-John E.Cavalca, LuciaNyman, DagCavuoto, Kara M.O’Sullivan, OrlaCayol, Jean-LucO’Brien, ClaireCelińska, EwelinaOchiai, YoshitsuguCentanni, ManuelaOchoa-Leyva, AdrianCerenius, LageO’Connell, CatherineCertad, GabrielaOechslin, FrankCervantes, MarcellaOggioni, MarcoCervino, GabrieleOgihara, JunChalker-Scott, LindaOh, Seung-YoonChami, GoyletteOhtomo, RyoChan, Edward D.Okamoto, ShigefumiChanda, AnindyaOlaru, Octavian TudorelChang, Yi-tangOliva, AlessandraCharpentier, XavierOliveira, IreneChase-Topping, MargoOlson, Jonathan W.Chaudhary, Dhiraj KumarOngerth, JerryChaudhuri, RoyOpitz, JörgChaves, Luis FernandoOrandi, SanazChaves, Susana RodriguesOrefice, IdaChelsky, DanielOresnik, IvanChen, Guang-YingOrlando, JulietaChen, GuijieOrlic, SandiChen, Jiann-ChuOrsini, MassimilianoChen, LongOsborne, NickChen, Pao-YangOsorio, CarlosChen, Shuen-EiÖstman, ÖrjanChen, XiaolinOstrowski, MartinChen, Yuh-shuenOtlewska, AnnaChen, ZhongOts, KatriChenoll, EmparOzkan, JeromeCheptsov, VladimirP. Sagona, AntoniaCheshenko, NataliaPace, FabioChiarelli, LaurentPachathundikandi, Suneesh KumarChiarini, LuigiPaduszynska, MalgorzataChifiruc, Mariana CarmenPagano, JosephChiou, Chien-ShunPanico, AntonioChistoserdova, LudmilaPantůček, RomanChochlakis, DimosthenisPapadelli, MarinaChoi, Jong MinPapagiannitsis, Costas C.Choi, Won HyungPapanikolaou, SeraphimChorianopoulos, NikosPapciak, DorotaChow, Franklin Wang-NgaiPapin, James F.Chronopoulou, MyrsiniPapoutsis, KonstantinosChrzanowski, ŁukaszPapp, JohnChua, BeeleePappas, JaniceChui, LindaPapp-Wallace, Krisztina M.Chul-sung, HuhParamythiotou, ElizabethCiacci, NagaiaParanhos-Baccalà, GlauciaCiani, MaurizioParisini, EmilioCieplik, FabianPark, Ki HwanCipak, LubosPark, Myeong SooCirés, SamuelPark, Soo JeCitterio, BarbaraPark, Young-SeoClark, Nicholas J.Parkar, Shanthi G.Clavijo-McCormick, AndreaParma, LucaClay, KeithPatakovaa, PetraClerc, PhilippePatarata, LuísClyne, MargueritePátek, MiroslavCoburn, Phillip S.Patel, MrudulaCogliati, MassimoPatelski, PiotrCohen, Jerry D.Paterson, Gavin K.Coldea, TeodoraPaterson, R. Russell M.Cole, BenjaminPathak, AshishCole, JeffPatini, RomeoColeman, JohnPatrauchan, Marianna A.Collett, AndrewPatrone, VaniaColmant, AgathePaul, Narayan ChandraColonna, BiancaPawar, KamleshColoretti, FabioPawar, ShrikantConnelly, SheilaPawlak, AleksandraConrads, GeorgPayne, MatthewConstantinos, PapagiannitsisPearce, CedricContaldo, NicolettaPearlman, Ronald EdwardContreras-Cornejo, Hexon AngelPeces, MiriamCoraça-Huber, Débora C.Peck, RonaldCordero-Bueso, GustavoPecyna, PaulinaCorfield, Anthony P.Pelagalli, AlessandraCorino, CarloPeng, MengfeiCorrea, MaríaPepi, MilvaCorreia Coelho, Ana CláudiaPeretz, AviCorsaro, CarmeloPerez Pascual, DavidCorte, LauraPerez, LaurentCorzo-Leon, DoraPérez-Gracia, María-TeresaCorzo-León, Dora E.Pérez-Montaño, FranciscoCostas, Amaya GarciaPerez-Rodríguez, MarthaCostea, Paul IgorPerng, Guey-ChuenCoulson, SamanthaPerricone, CarloCox, Jonathan A.G.Perrin, ElenaCoyne, Mark S.Perujo, NuriaCrawford, Emily D.Petar, PujićCrawford, LindseyPeters, BrandilynCrecchio, CarminePeterson, BrandonCreuzenet, CarolePeterson, Eric CharlesCrnkovic, AnaPetreaca, Ruben C.Crook, BrianPetruccioli, MaurizioCrossan, ClairePfaff, Alexander W.Crossman, LisaPfliegler, Walter P.Cruaud, PerrinePhalen, DavidCuadra, GiancarloPhillips, Gregory J.Cukrowska, BoženaPianciola, LuisCurtin, ChristopherPicchianti-Diamanti, AndreaCzyżowska, AgataPickett, AndyD. Bortner, CarlPieckova, ElenaD’Apice, LucianaPiepenbrink, KurtDa Costa, João PintoPierce, EllenDaems, DevinPiercey-Normore, MicheleDaghio, MatteoPiñeiro, LuisDahl, Jan-UlrikPires, RicardoDaigle, FrancePisarenko, Sergey V.Dalloul, RamiPischke, SvenDame, RemusPitre, FrédéricDang, ZhiPittau, MarcoDanziger, Larry HPlacido, JerssonDarre, Michael J.Plassard, ClaudeDas Gupta, SanatanPlaza-Díaz, JulioDas, SauravPlessas, StavrosDas, SubhaPletzer, DanielDas, UndurtiPlochberger, BirgitDasch, Gregory A.Polissi, AlessandraDatta, KausikPolkowska, ZanetaDatta, RahulPoltronieri, PalmiroDavenport, Emily R.Poma, PaolaDavid, MichaelPonton, FleurDavido, BenjaminPopova, AntoanetaDavis, JamesPotekhin, AlexeyDavydenko, KaterynaPotnis, NehaDe Francesco, GiovanniPoudel, BarunDe Godoy, Maria R. C.Poyntner, CarolineDe Lima, Valéria Marçal FelixPrabakaran, MookkanDe Soet, Johnannes JacobPrado-Cabrero, AlfonsoDe Swart, RikPratte, ZoeDegli Esposti, MauroPrearo, MarinoDelbarre Ladrat, ChristinePrescott, John F.Dell’Oste, ValentinaPresentato, AlessandroDenkova-Kostova, RositsaPrice, Christopher T.D.Deptula, PaulinaPrice, PatriciaDeryabina, Yu I.Price-Carter, MarianDesai, JigarPrigione, ValeriaDescombes, PatrickProdanovic, RadivojeDeshpande, ShayuProestos, CharalamposDevirgiliis, ChiaraProft, ThomasDevkota, Hari PrasadPruszyńska-Oszmałek, EwaDewasme, LaurentPrzybilski, RitaDhakal, SantoshPsaroulaki, AnnaDi Domenico, Enea GinoPszczółkowska, AgnieszkaDi Ioia, DianaPuchkov, EvgenyDi Onofrio, ValeriaPuig, SergiDi Pietro, MarissaPuolakkainen, MirjaDi Stasio, DarioPurkamo, LottaDiaz, Julia M.Pushko, Dr. PeterDib, Julián RafaelPyzik, AdamDieser, MarkusQuilez, JoaquinDieterich, WalburgaQuinn, JanetDillman, AdlerRabbani, GulamDillon, Jo-Anne R.Radoshevich, LillianaDimitrellou, DimitraRaheem, BamideleDinardo, FrancescaRahman, FarooqDing, LinRahman, Masmudur M.Ding, QingbaoRai, NavneetDionisio, GiuseppeRajaiya, JayaDiretto, GianfrancoRam, Angia Sriram PradeepDix, RichardRamachandran, VinoyDolfing, JanRamos Morales, FranciscoDomagała, SławomirRamos-Vivas, JoséDomínguez, ÁngelRanganathan, NatarajanDominiak, MarzenaRaquel, BecerrilDonald, Claire L.Rastelli, EugenioDonaldson, JanetRatinier, MaximeDong, XiyangRatkowsky, DavidDonnarumma, GiovannaRauwel, ErwanDore, Maria PinaRaval, YashDorea, CaetanoRawla, PrashanthDorrell, Richard G.Rayan, AnwarDosoky, NouraReale, AnnaDostalek, PavelReddy, PriyankaDoulgeraki, AgapiReed, David W.Downing, TimReed, Kevin F.M.Doyle, Evelyn M.Reisch, Christopher R.Dozois, CharlesRemacle, ClaireDrevets, Douglas A.Renaudie, JohanDreyfus, GeorgesRenčiuk, DanielDrosinos, EleutheriosRepetto, OmbrettaDrown, Devin M.Requicha, JoaoDu, YongleRestivo, Francesco MariaDu, ZhibinRestrepo, MarcosDuarte, AmilcarRety, StephaneDubovskiey, IvanReuter, TimDucatez, MarietteRey, LuisDucluzeau, Anne LiseRhayat, LamyaDuffy, Fergal JRho, Hoon SukDurgo, KsenijaRho, HyungminDuring, EmmanuelRial, RaquelĐurović, SašaRibeiro, ApoenaDutartre, HélèneRiccardo, OrusaDutta, SanjuctaRichard, PeterDziedzic, ArkadiuszRichner, Justin M.Dziewit, LukaszRiedel, Sebastian L.Eboigbodin, KevinRikkinen, JoukoEconomou, VangelisRinninella, EmanueleEda, ShigetoshiRios-Covian, DavidEdgcomb, VirgniaRipari, ValeryEdlich, FrankRishpon, JudithEdwards, Simon G.Riska, PaulEfremenkova, Olga VRiva, AntonioEhrhardt, ChristopherRivas, GermanEichhorn, IngaRivilla, RafaelEl Kasmi, FaridRizzo, Peter J.Elbadry, MahaRoane, TimberleyEliopoulos, Panagiotis A.Robinson, RichardElleuche, SkanderRobinson, SeriElse, Terry AnnRocha, JaquelineEnache, Mădălin-IancuRodrigues, Célia F.Enache-Angoulvant, AdelaRodriguez, HectorEnderle, RasmusRodriguez, PatriciaEngelberg, DavidRodríguez-Jerez, José JuanEngelmann, IlkaRodríguez-López, PedroEnglezos, VasileiosRodriguez-Palacios, AlexanderEnguita, Francisco J.Rodríguez-Ruano, Sonia MaríaErauso, GaelRodriguez-Rubio, LorenaErben, MauricioRoeva, OlympiaEsadze, AlexandreRomano Spica, VincenzoEscott, CarlosRomano, NiclaEscuret, VanessaRoques, ChristineEspadaler Mazo, JordiRoscini, LucaEspasa, MateuRosenberg, MerilinEspinel-Ingroff, AnaRoss, IanEspinosa Padrón, ManuelRossi, FrancaEsteve-Romero, JosepRossi, FrancaEstevinho, Leticia M.Roszczenko-Jasinska, PaulaEszterbauer, EditRoth, MichaelEtxebeste, OierRoy, DenisEvans, Franklin D.Roy, JulienEvilia, CarynRuaro, BarbaraEyice-Broadbent, OzgeRubio, LuisEyssautier, StéphanieRubio, MyriamFagerquist, CliftonRuffell, SarahFaia, Arlete MendesRuiz-Moyano, SantiagoFalade, TitilayoRusso, PasqualeFalconer, IanRusso, RositaFaleiro, Maria LeonorRyan, MichaelFalkinham III, Joseph O.Ryan, Michael P.Fang, FrancisRybczyńska-Tkaczyk, KamilaFanizzi, Francesco PaoloRychlik, IvanFarber, MatthewSaadeldin, IslamFarcasanu, Ileana C.Sabella, ErikaFarnell, Morgan B.Sabet, ShereenFarrar, KerrieSabino, RaquelFaust, KarolineSadigh, YasharFavier, Anne-LaureSadowska, BeataFeliciano, JoanaSaelao, PerotFeniouk, BorisSaelens, JosephFeodorova, Valentina AŠafarič, LukaFerdy, Jean-BaptisteSaggese, AnellaFernández-Ochoa, ÁlvaroSaitoh, KenjiFerrante, AntonioSalaheen, SerajusFerreira, JorgeSalamon, SylwiaFerreira, Manuel MarquesSaleh, MonaFerrigo, DavideSalwan, RichaFetissov, Sergueï O.Sammi, ShreeshFettig, ChrisSánchez Torres, PalomaFijan, SabinaSanchez-Barrios, AndreaFiliatrault, MelanieSánchez-Campos, SoniaFine, James BurkeSánchez-Porro, CristinaFinetti-Sialer, Mariella MatildeSancineto, LucaFiorillo, LucaSandalakis, VassiliosFischer, Egil Andreas JoorSanders, Mary EllenFischer, NicoleSandle, TimFitzpatrick, DavidSannino, FilomenaFlach, Carl-FredrikSanso, Andrea MarielFlorent, IsabelleSantander, JavierFloris, RosannaSantos, JeffersonFlynn, RobinSantos, RicardoFlythe, MichaelSarbini, ShahrulFonseca, FilomenaSartini, MarinaForbey, Jennifer S.Sasase, TomohikoFord, Timothy E.Sass, HenrikForster, IanSassera, DavideFoster, TimSato, HiroshiFouillaud, MireilleSato, TakuichiFouladkhah, AliyarSato, Trey KFoysal, Md JavedSaurav, KumarFrade, SaraSavion, NaphtaliFragkou, Paraskevi C.Sawa, HirofumiFranchi, ElisabettaSaxena, VikasFranco, Carlos M.Scarlett, GarryFrançois Pouchus, YvesSchaaper, RoelFrasson, IlariaSchaffner, DonaldFredriksson-Ahomaa, MariaSchalk, Isabelle J.Freeman, Christopher JSchallmey, AnettFreimoser, FlorianSchaub, Günter A.Freitas-Almeida, Angela CorreaSchauss, AlexanderFroissart, RémyScheffers, SanderFu, FuyouSchippa, SerenaFu, PengSchlusselhuber, MargotFunari, RiccardoSchmidt, RebeccaFurneri, Pio MariaSchmidt-Heydt, MarkusFurukawa, KenjiSchneider, BarbaraFurukawa, KensukeSchoenle, AlexandraFuruse, YukiSchoepp-Cothenet, BarbaraGabrić, DraganaScholte, Florine E.M.Gagiu, ValeriaScholzen, AnjaGajardo, GonzaloSchroeder, Bjoern O.Gajdács, MárióSchughart, KlausGalachyants, Agnia D.Schwaderer, AndrewGalán-Sánchez, FátimaSchwan, WilliamGałązka, AnnaSchwarz, PatrickGalindo-Villegas, JorgeSchweigkofler, WolfgangGallagher, Daniel L.Schwiertz, AndreasGalvez, Eva M.Sclavi, BiancaGama, JoaoScotti, RiccardoGambley, Cherie F.Scullion, JohnGantner, MagdalenaScully, SeanGanusov, VitalySechi, Leonardo A.Gao, BeiŠegvić Klarić, MajaGarbayo, InésSękara, AgnieszkaGarcía Suárez, PilarSekse, CamillaGarcia, CyrielleSelan, LauraGarcia-Descalzo, LauraSeligmann, HerveGarcía-Díez, JuanSellam, AdnaneGardlik, RomanSelva, StevenGargari, GiorgioSengupta, SonaliGarner, BiancaSeo, Ho SeongGarnica, SigisfredoSeppey, ChristopheGarofalo, MariangelaSeravalli, JavierGarrido-Sanz, DanielSerra, Claudia R.Gasiūnas, GiedriusSevilimedu Veeravalli, SathyanarayananGautam, AartiShahid, SalmanGauvin-Bialecki, AnnaShakya, Viplendra P.S.Gavelis, Gregory S.Shamoun, SimonGeml, JózsefShanks, RobGenilloud, OlgaShao, QiuyanGharsallah, HoudaSharma, Narinder KumarGheorghiu, EugenSharma, ParmodhGhiasi, HomayonSharov, AndreyGhigo, EricShaw, AndrewGhobadi, SajjadShcherbakova, ViktoriaGhosh, MrinmoyShemesh, MosheGhosh, SumitShen, GarryGiannoni, FedericoShetty, Amol C.Giannuzzi, LedaShetty, Sudarshan A.Gianotti, AndreaShinde, TanviGiaveno, AlejandraShoguchi, EiichiGibson, Deanna L.Shokal, UpasanaGieg, LisaShrestha, BhushanGil, RosarioShymanovich, TatsianaGill, HarsharnSiddappa, ByrareddyGil-Martinez, MartaSidorenko, SergeyGiordani, PaoloSierra, Josep M.Giordano, LuanaSilar, PhilippeGiri, SibSiltz, Lauren A.Glazunova, Olga A.Silva, Christopher J.Glockner, KarlSilvan, Jose ManuelGlorieux, GrietSilverstein, RachelGnanasundram, Sivakumar VadivelSimões, Marta FilipaGodbold, Douglas LSimonin, YannickGodfrey, Henry P.Singh, Atul KumarGolemi-Kotra, DasantilaSingh, Dr. KhushwantGolubkina, Nadezhda A.Singh, HarinderGomes, SóniaSingh, PallaviGomez De Agüero, MercedesSingh, RajbirGómez, FernandoSingh, SukhvinderGomez-Gallego, CarlosSinha, DebottamGonda, SándorSiniscalco, DarioGong, LiminSioutopoulos, DimitriosGoñi, PilarSip, AnnaGonzalez Viejo, ClaudiaSipiczki, MatthiasGonzalez, GabrielSipkema, DetmerGonzalez, Juan M.Sivaprakasam, SathishGonzález-Candelas, LuisSkidmore, MarkGonzález-Fandos, María ElenaSklodowska, AleksandraGonzález-Siso, María-IsabelSkraban, JureGonzález-Soltero, RocíoSlaby, BeateGoodman, Alan G.Slamova, KristynaGornik, SebastianSlamovits, ClaudioGorres, Kelly L.Slootweg, Erik JGorwa-Grauslund, Marie FrancoiseSmedile, FrancescoGórzyńska, KarolinaSmith, StephenGossman, ToniSnyder, LoriGöttfert, MichaelSoares Silva, Isabel JoaoGournas, ChristosSockett, LizGoyal, RajniSolano, FranciscoGozzo, FrancoSolyanikova, Inna P.Grace-Farfaglia, PatriciaSommers, PacificaGragg, Sara E.Sonah, HumiraGrant, IreneSørensen, Jens LauridsGrąt, MichałSosio, MargheritaGreene, AnnelSousa, AngelaGreenwood, Michael T.Sousa, Maria JoãoGreetham, DarrenSpadaro, SavinoGrgurina, IngeborgSpiers, AndrewGrishin, Alexander V.Spina, FedericaGrisoli, PietroSplichal, IgorGrosse, StephanSplichalova, AllaGrouzdev, Denis S.Springael, DirkGruber, StephanSquartini, AndreaGrywalska, EwelinaSreevatsan, SrinandGrządziel, JarosławStan, MirunaGrzebyk, DanielStana, AncaGrzesiak, JakubStanford, KimGrzybek, MaciejStanish, LeeGuarino, FrancescoStasiak-Różańska, LidiaGuerreiro, InêsStauff, DevinGuldimann, ClaudiaStefani, FranckGundamaraju, RohitStefanini, IreneGupta, CharuStephan, RogerGUPTA, KULDEEPStevens, DavidGupta, VijaiStevens, David ColeGustafson, John E.Stevens, Joanne M.Guzow-Krzemińska, BeataStevenson, KarenGweon, Hyun SoonStevenson, RobertGyawali, RabinStewart, PhilHabib, IhabStingl, Ulrich (Uli)Hackmann, TimothySt-Pierre, BenoitHać-Szymańczuk, ElżbietaStrati, KaterinaHafner, Louise MarieStraube, EberhardHahn, MartinStricker, RaphaelHalos, LenaigStringlis, IoannisHan, QifengStudenik, SandraHara, HirofumiStuhr, MarleenHarada, SoheiStumpff, FriederikeHarapan, HarapanSturtevant, JoyHarazny, Joanna M.Suchanek, M.Hardwidge, PhilipSugawara, AkihiroHarkin, Kenneth R.Sukdeo, NicoleHarrison, Joshua G.Sun, ChangpoHauge, Sigrun J.Sun, KunfengHaverkamp, ThomasSun, XiaofeiHay, RodSun, XiaomingHayes, FinbarrSun, XingminHeider, JohannSundheim, LeifHeinonen, Krista M.Sungthong, RungrochHelena, Guasch PadróSunna, AnwarHémadi, MiryanaSvircev, Antonet M.Henderson, RitaSwidsinski, AlexanderHenkel, JaninSzafranek-Nakonieczna, AnnaHernández-León, AlejandroSzewczyk, KatarzynaHernández-Rodríguez, CésarSzweda, PiotrHESLER, LOUIS S.Szymańska, JolantaHeyndrickx, MarcTai, WanboHiejima, YusukeTall, Ben D.Hietala, AriTalon, RégineHiggins, N.Tamaru, YutakaHiguchi, TomihikoTannous, JoannaHikal, WafaaTansey, JohnHill, AnnieTanyeri, MelikhanHille, KatjaTaoka, YousukeHindra, HindraTartera, CarmenHinkula, JormaTashima, ToshihikoHiraishi, AkiraTaverniti, ValentinaHirsch, Alec J.Taylor, MatthewHiruma, KeiTedbury, PhilipHoang, Nam VTeixeira, MiguelHoel, SunnivaTelle-Hansen, Vibeke H.Hoenigl, MartinTellez-Isaias, GuillermoHogberg, NilsTemesgen, Tamirat TeferaHolland, MartinTengs, TorsteinHollsberg, PerTeramoto, TadahisaHong, Su-HyungTeras, RihoHönig, VáclavTerova, GencianaHorhat, Florin GeorgeTerpou, AntoniaHosomi, RyotaTerzi, ValeriaHou, HarveyTesh, VernonHouldcroft, CharlotteTfaily, MalakHowarth, GordonThakur, AneeshHrdý, JiříThapa, SantoshHu, H.-D.Tharmalingam, NagendranHu, JunhuiThekkiniath, JoseHuang, KaiThielen, BethHuang, LinfengThies, StephanHuang, MianThomas, María del MarHuang, XinTirloni, EricaHuang, ZhijianTiscar, Pietro G.Huang, Zuyi (Jacky)Tiwari, Rakesh K.Hughes, DiarmaidTkacz, AndrzejHulme, JohnTkavc, RokHünigen, HanaTofalo, RosannaHurtado, AnaToiu, AncaHybiske, KevinTomaso, HerbertHynes, Michael F.Toovey, StephenIbrahim, Mohamed AliToranzo, Alicia E.Iebba, ValerioToribio-Mateas, MiguelIglesias, Alberto Á.Torlapati, JagadishIlan, MichaTörn, CarinaImhoff, JohannesToro, Mario DamianoImperial, JuanTorres Barcelo, ClaraIndugu, NagarajuToshchakov, StepanIngram-Smith, CherylTotta, PierangelaInoue, DaisukeToyoma, TadashiIorizzo, MassimoTrautwein-Schult, AnkeIpharraguerre, IgnacioTrček, JanjaIrie, JunichiroTreu, RolandIsakova-Sivak, IrinaTreusch, Alexander H.Isokpehi, RaphaelTripathi, Jitendra KumarIstrate, Irina AuraTripathi, Siddharth KaushalIwasaki, KazuhiroTrivedi, PankajIzard, JacquesTrögl, JosefIzasa, HisashiTrubl, GarethIzzo, AngeloTrzaskowska, MonikaJackson, SimonTsai, An-YiJacob, PaulTse-Dinh, Yuk-ChingJafari, Seid MahdiTsiaoussis, JohnJagadeesan, BalamuruganTsirigotis, PanagiotisJagus, RosemaryTsironi, TheofaniaJahidul, IslamTsuchida, SachioJain, VaibhavTsuji, MasaharuJaiswal, Ashvin R.Tu, PengchengJakobsen, MogensTuanyok, ApichaiJames, Euan KevinTuchscherr, LorenaJameson, EleanorTucker, JamesJanczukowicz, WojciechTulunoglu, IbrahimJanga, Srikanth ReddyTuovinen, OlliJarocki, VeronicaTurchetti, BenedettaJarvis, SusanTurnau, KatarzynaJarzebski, MaciejTurner, Raymond J.Jehmlich, NicoTurroni, SilviaJelocnik, MartinaTurton, JaneJia, KailiangTutino, Maria LuisaJiang, YuemingTyson, GregoryJohnson, Shannon L.Tyson, JessJordao, LuisaUchio, EiichiJose Manuel, Jose ManuelUgalde, Juan E.Joseph, SusanUmemura, MycoJoshi, AmitUmerska, AnitaJosset, LaurenceUnderwood, Keith R.Juottonen, HeliUnković, NikolaJurado, M.M.Unnikrishnan, MeeraKaartokallio, HermanniUnno, TatsuyaKabir, NiamulUpadhyay, AbhinavKacaniová, MiroslavaUpadhyay, ChitraKaczorek, EwaUpdegrove, Taylor B.Kaczorowski, TadeuszUrabe, HiroakiKalatzis, Panos G.Urbańczyk-Lipkowska, ZofiaKaldhone, PravinUrraca, Javier L.Kalinowska, KrystynaUsui, TatsufumiKalra, DivyaVahjen, WilfriedKalyuzhnaya, MarinaValdes-Lopez, OswaldoKamalanathan, ManojValéntín, EulogioKamczyc, JacekValentine, Rudy J.Kamzolova, SvetlanaValiadi, MarthaKanda, TatsuoValitutti, FrancescoKanda, TeruVallance, BruceKandylis, PanagiotisVallesi, AdrianaKaneko, JunVallini, GiovanniKang, Sang SunValls, MarcKang, SanghoonValm, Alex M.Kapanidis, AchillefsVamanu, EmanuelKarachaliou, MariannaVan Campenhout, LeenKaradjian, GregoryVan Der Nest, Magriet AKarahan, H. EnisVan Domselaar, GaryKarakula-Juchnowicz, HannaVan Etten, JamesKarayanni, HeraVan Lanen, StevenKarpe, Avinash V.Van, Thi Thu HaoKarpiński, Tomasz MVandenkoornhuyse, PhilippeKartheek, AnekellaVarani, StefaniaKarus, AvoVarzakas, TheodorosKashtanova, DariaVasse, MarieKasperski, AndrzejVautier, SimonKaszowska, MartaVaz-Moreira, IvoneKato, Takamitsu AVazquez Belda, BeatrizKatouli, MohammadVázquez Sánchez, DanielKaufer, BenediktVemuri, RavichandraKaushik, PrashantVenkannagari, HarikanthKavita, KumariVenkatesh, SiddarthKawai, FusakoVerdenelli, CristinaKaye, StephenVerma, SagunaKebbi-Beghdadi, CaroleVerma, Satish KKeyel, Peter A.Verma, SmritiKhadempour, LilyVerni, MichelaKhan, Atif AliVeyron-Churlet, RomainKhan, NaimVidal, RebecaKhanipov, KamilVidovic, SinisaKhatri, Vishal KishorVieira, SelmaKieffer, NicolasVijgenboom, ErikKieliszek, MarekVilain, SébastienKietäväinen, RiikkaVílchez-Vargas, RamiroKim, Byoung SikVilela, AliceKim, ChyerVilibić-Čavlek, TatjanaKim, Dal YoungVilla, Tomas G.Kim, HoonVimberg, VladimirKim, HoyeunViñas, MiguelKim, Hyeun BumVincent, AntonyKim, Hyung-LaeVincze, SzilviaKim, Ji HyungVirok, Dezső P.Kim, Min-SukVitetta, LuisKim, YunjeongVladimir, KalininKireev, IgorVlainić, JosipaKirienko, NatashaVon Bergen, MartinKirschner, DeniseVotypka, JanKishida, MasaoVuotto, ClaudiaKitazawa, TakioWajs-Bonikowska, AnnaKjelland, VivianWałecka-Zacharska, EwaKlassen, ViktorWaleron, KrzysztofKlewicka, ElzbietaWallace, Nicholas A.Klimina, Ksenia M.Walsh, AaronKmiec, Eric B.Walther, GritKniel, KalmiaWalther-Antonio, MarinaKnoshaug, EricWan, ShibiaoKobayashi, KazuoWang, Chao-MinKobayashi, NobumichiWang, GuorongKocsis, BelaWang, JuexinKogut, Michael H.Wang, JunyeKoh, HyunwookWang, KaiKokkinakis, EmmanouilWang, KankanKokkinos, NikolaosWang, LeyiKokou, FotiniWang, Robert Y.-L.Koksharova, Olga A.Wang, ShihuaKoller, MartinWang, ShueKollipara, AvinashWang, ZunyiKolobov, Alexandr AlexandrovichWareth, GamalKomaki, HisayukiWasinger, Valerie C.Konjar, ŠpelaWatanabe, MihoKonno, NaotakeWeber, J. MarkKontsevaya, IrinaWegner, Carl-EricKooij, Pepijn W.Węgrzyn, MichałKordalewska, MilenaWeinhold, ArneKormas, Konstantinos Ar.Wemheuer, BerndKorneev, DenisWemheuer, FranziskaKornitzer, DanielWen, Chiu-MingKosakyan, AnushWendisch, Volker F.Kostecka, MalgorzataWerner, JohannesKot, AnnaWessely-Szponder, JoannaKotlowski, RomanWestra, Edze R.Kounatidis, IliasWhitehouse-Tedd, KatherineKoutelidakis, Antonios E.Whitman, Christian P.Kovacs, RenatoWhitworth, David E.Kovatcheva-Datchary, PetiaWidemann, EmilieKowalkowski, TomaszWiktor, ArturKozak, BartoszWiley, Clayton A.Kozlowski, LukaszWilharm, GottfriedKreuzer-Redmer, SusanneWisselink, HenkKrier, FrançoisWnuk, MaciejKrisztián, BaloghWohlrab, SylkeKrivoruchko, AnastasiiaWojkowska-Mach, JadwigaKrol, EwelinaWolf, YuriKroukamp, HeinrichWolińska, AgnieszkaKrstanović, VinkoWong, AdamKrutova, MarcelaWong, Chi-MingKu, SeockmoWong, JosephKubota, KeiichiWöstemeyer, JohannesKucharíková, SoňaWu, ChaoKucharska, KarolinaXi, WeixianKudłak, BłażejXiao, XiaKudva, Indira T.Xie, JieKulakovskaya, Tatiana V.Xie, MouzheKulhánek, MartinXie, WankunKumar, AmitXing, JunjiKumar, PradeepXu, JianKumar, RohitashwYablonski, GaliaKumar, SujeetYadav, PramodKumar-Singh, SamirYagüe, PaulaKunda, NiteshYahr, RebeccaKuroda, MasashiYamada, ShuheiKurosawa, NorioYamamoto, NaomichiKurtböke, IpekYamamura, HiroshiKurtti, TimothyYamanaka, TakashiKushkevych, IvanYamayoshi, SeiyaKuzdraliński, AdamYan, AixinKuźniar, AgnieszkaYan, JingKwok, Hang Fai (Henry)Yan, XiaKyoung-soon, JangYanagisawa, NaokoLaanto, ElinaYang, Feng-LingLagali, NeilYang, Hung-ChihLai, Chih-ChengYang, Seung HwanLanga, SusanaYao, ZhenqiangLanternier, FannyYaoita, YasunoriLaPointe, GisèleYau, Yunki Y.Lappa, IliadaYeh, Jun-JunLara, EnriqueYeldandi, VijayLaranjo, MartaYilmaz, BahtiyarLarkin, Robert P.Yip, Bon HamLarsen, Ray A.Yokawa, KenLarussa, TizianaYokosyo, KengoLastauskienė, EglėYoshida, MakotoLavergne, Rose-AnneYoshinaga, MasafumiLavin, MattYoungblut, NicholasLawson, PaulYu, Feiqiao BrianLax, SimonYu, GangLeavitt, SteveYuan, ZuanningLebar, MatthewYukl, Erik T.Lee, Heung KyuYumoto, IsaoLee, Je ChulYun, Cheol-WonLee, Jung RoYurimoto, HiroyaLee, Moo-SeungZago, MiriamLee, Myung-ShikZaini, PauloLee, Po-Heng (Henry)Zaiter, AliLee, Shee EunZalewska, AnnaLee, Sung-EunZambrano-Wilson, Maria ClemenciaLee, Wan-KyuZara, GiacomoLeewis, Mary-CathrineZawierucha, KrzysztofLeitão, AnaZdarta, AgataLeitão, Jorge H.Zeleny, ReinhardLeitch, CarolZendo, TakeshiLemercier, GillesZeng, JILemfack, Marie ChantalZeng, MelodyLetek, MichalZhang, MingLeung, Chung Yin (Joey)Zhang, RuiyongLevy, StevenZhang, TingLi, ChunfengZhao, JingLi, DongmeiZhao, TongkeLi, Fu-shuangZhao, XihongLi, SimengZhou, BangjunLi, YihangZhou, MiLi, ZhongweiZhu, ChrisLiao, ChenZhu, JingenLiao, Chien-SenZhu, JunjieLiao, YucaiZhu, LuchangLiao, YuxingZhu, QifanLiapikou, AdamanthiaZhuang, GuangchaoLibault, MarcZhukov, Vladimir A.Liebman, MichaelZiegler, MarenLiechti, George W.Zielenkiewicz, UrszulaLim, Young-WoonZimba, PaulLima, Bruno P.Ziogas, VasileiosLin, Asiyah (Yu)Zmora, PawelLin, Ching-HsuanZou, WeiLin, HuaiyingZuber, PeterLin, Jiun-NongZuo, RanLin, NancyZwittink, RomyLin, Ying-Chi


